# Osteochondral Tissue Chip Derived From iPSCs: Modeling OA Pathologies and Testing Drugs

**DOI:** 10.3389/fbioe.2019.00411

**Published:** 2019-12-17

**Authors:** Zixuan Lin, Zhong Li, Eileen N. Li, Xinyu Li, Colin J. Del Duke, He Shen, Tingjun Hao, Benjamen O'Donnell, Bruce A. Bunnell, Stuart B. Goodman, Peter G. Alexander, Rocky S. Tuan, Hang Lin

**Affiliations:** ^1^Department of Orthopaedic Surgery, Center for Cellular and Molecular Engineering, University of Pittsburgh School of Medicine, Pittsburgh, PA, United States; ^2^Xiangya School of Medicine, Central South University, Changsha, China; ^3^Department of Bioengineering, University of Pittsburgh Swanson School of Engineering, Pittsburgh, PA, United States; ^4^Department of Pharmacology, Center for Stem Cell Research, Tulane University School of Medicine, New Orleans, LA, United States; ^5^Department of Orthopaedic Surgery and Bioengineering, Stanford University, Stanford, CA, United States; ^6^McGowan Institute for Regenerative Medicine, University of Pittsburgh School of Medicine, Pittsburgh, PA, United States

**Keywords:** iPSCs, DMOADs, tissue chip, osteoarthritis, Celecoxib

## Abstract

Osteoarthritis (OA) is a chronic disease mainly characterized by degenerative changes in cartilage, but other joint elements such as bone are also affected. To date, there are no disease-modifying OA drugs (DMOADs), owing in part to a deficiency of current models in simulating OA pathologies and etiologies in humans. In this study, we aimed to develop microphysiological osteochondral (OC) tissue chips derived from human induced pluripotent stem cells (iPSCs) to model the pathologies of OA. We first induced iPSCs into mesenchymal progenitor cells (iMPCs) and optimized the chondro- and osteo-inductive conditions for iMPCs. Then iMPCs were encapsulated into photocrosslinked gelatin scaffolds and cultured within a dual-flow bioreactor, in which the top stream was chondrogenic medium and the bottom stream was osteogenic medium. After 28 days of differentiation, OC tissue chips were successfully generated and phenotypes were confirmed by real time RT-PCR and histology. To create an OA model, interleukin-1β (IL-1β) was used to challenge the cartilage component for 7 days. While under control conditions, the bone tissue promoted chondrogenesis and suppressed chondrocyte terminal differentiation of the overlying chondral tissue. Under conditions modeling OA, the bone tissue accelerated the degradation of chondral tissue which is likely via the production of catabolic and inflammatory cytokines. These findings suggest active functional crosstalk between the bone and cartilage tissue components in the OC tissue chip under both normal and pathologic conditions. Finally, a selective COX-2 inhibitor commonly prescribed drug for OA, Celecoxib, was shown to downregulate the expression of catabolic and proinflammatory cytokines in the OA model, demonstrating the utility of the OC tissue chip model for drug screening. In summary, the iPSC-derived OC tissue chip developed in this study represents a high-throughput platform applicable for modeling OA and for the screening and testing of candidate DMOADs.

## Introduction

Osteoarthritis (OA) is a common debilitating disease characterized by the progressive degeneration of the articular cartilage and remodeling of other joint elements. Global statistics revealed that over 100 million people worldwide suffer from OA (Bhatia et al., 2013). In the United States alone, 10% of men and 13% of women over 60 years-old have been diagnosed with knee OA (Zhang and Jordan, 2010). OA causes pain and impairs mobility, giving rise to a significant societal and economic burden (Dibonaventura et al., 2011). Currently, the pathogenesis of OA is not clearly understood. Furthermore, there are no FDA-approved disease-modifying OA drugs (DMOADs) for treatment. This unmet clinical need is due, in part, to the lack of robust models to precisely mimic the pathology of OA in humans. Although animal models are often used in biomedical research (Rosenthal and Brown, 2007), the translation potential of these animal models to human conditions is often low (Shanks et al., 2009; Seok et al., 2013). *In vitro* models, commonly conventional 2-dimensional chondrocyte cultures, even with the use of human cells, do not adequately reflect the complexities of cell-cell interactions in the 3-dimensional tissue context, thus neglecting the whole joint disease concept of OA (Mosig, 2017). The incongruence between *in vitro* models of OA and *in vivo* pathogenesis, and the potentially different disease mechanisms between human and model animals, together contribute to make the etiology and pathology of OA still speculative.

To address these issues, we proposed the creation of a physiologically and anatomically relevant *in vitro* model of defined tissue-specific functions with human cells to better study and understand the pathogenesis of OA. Cartilage and bone represent the two principal components in the articular joint affected by OA and display different structural and functional properties. Articular cartilage is a highly hydrated viscoelastic structure, rich in collagen type II, and sulfated proteoglycans (Sophia Fox et al., 2009). In contrast, bone is a vascularized tissue mainly comprised of a stiff interstitial matrix of predominantly hydroxyapatite-bound collagen type I (Le et al., 2017). Cartilage and bone are in direct contact at the OC junction (OCJ), which also serves as a locus for biological and biomechanical crosstalk between cartilage and bone (Yuan et al., 2014; Findlay and Kuliwaba, 2016). Different types of molecules can diffuse through the bone-cartilage interface and this permeability is elevated with the progression of OA (Hwang et al., 2008; Pan et al., 2009). Specifically, the factors released from subchondral bone can induce cartilage degradation under OA conditions, and *vice versa* (Sharma et al., 2013). Therefore, both bone and cartilage should be considered as an integrated OC unit in the study of OA pathogenesis and the development of DMOADs.

Our laboratory has previously developed an *in vitro* OC model using human bone marrow-derived adult mesenchymal stem cells (MSCs) (Lin et al., 2014b). However, there are several limitations associated with the use of MSCs. Importantly, MSCs exhibit diminished differentiation potential associated with increased culture passage and have finite expansion capacity, thus limiting the number of total cells available for the generation of OC tissue chips. Therefore, MSC-derived OC tissues lack feasibility for future high throughput drug screening. Furthermore, donor-to-donor variability of MSCs may result in batch-to-batch difference of the engineered OC tissue, thus compromising reproducibility. As an alternative to MSCs, induced pluripotent stem cells (iPSCs) have, theoretically, unlimited proliferative capacity and chondrogenic/osteogenic potential. In one of our recent studies, iPSCs were induced to an MSC-like state, referred to as iPSC-derived mesenchymal progenitor cells (iMPCs). Similar to primary MSCs, the iMPCs possessed potent chondrogenic and osteogenic differentiation capabilities upon appropriate stimulation (Diederichs and Tuan, 2014).

In this study, we first optimized the procedure to induce chondrogenesis and osteogenesis of iMPCs. Then the iMPCs were encapsulated within gelatin scaffolds and cultured in the dual-flow bioreactor, which was previously developed in our laboratory (Lin et al., 2014b). The OC tissue generated from iMPCs were characterized after 28 days of differentiation. To examine the potential effect of bone on cartilage health, cartilage only constructs were also created. The generation of an OA model in an iPSC-derived OC tissue chip followed a previously established method (Lin et al., 2014b), in which interleukin-1β (IL-1β) was introduced to the medium flow of the chondral side. Finally, Celecoxib, a widely prescribed non-steroidal anti-inflammatory drug (NSAID), was tested in the system. In this study, the formation of cartilage tissue from iMPCs as well as the responses of the engineered cartilage to IL-1β and Celecoxib, with or without the presence of a bone component, were examined. Findings from this study provide support and details on the functional crosstalk between bone and cartilage under normal and OA conditions.

## Materials and Methods

### Generation and Expansion of Induced Pluripotent Stem Cells (iPSCs)

Human iPSCs was generated by reprogramming primary human bone marrow-derived MSCs (hBM-MSCs) as previously described (Diederichs and Tuan, 2014). The hBM-MSCs were isolated from a 48 year-old donor. We obtained hBM-MSCs from the femoral heads of patients undergoing total hip replacement with the Institutional Review Board's approval (University of Washington, Seattle, WA). The phenotype and differentiation capacity of iPSCs have been reported in a previous study (Diederichs and Tuan, 2014). The expansion of iPSCs were carried out according to standard protocol. Briefly, vitronectin XF (Stemcell Technologies, Vancouver, CA) was used to coat the culture plate before seeding of iPSCs. iPSCs were then fed with TeSR1 medium (Stemcell Technologies, Vancouver, CA). The medium was changed daily and cells were passaged at 80–90% confluency.

### Differentiation of iMPCs From iPSCs

iMPCs were generated from iPSCs using a protocol established in our lab (Diederichs and Tuan, 2014). When iPSCs reached 80% confluency, expansion medium was replaced with STEMdiff™-ACF Mesenchymal Induction Medium (Stemcell Technologies) for 3 days. Afterwards, MesenCult™-ACF Plus Medium (Stemcell Technologies) was used. On day 6, differentiated cells were detached and re-plated onto flasks that were pre-coated with Animal Component-Free Cell Attachment Substrate (Stemcell Technologies). The MesenCult™-ACF Plus Medium was used to expand these cells to 80% confluency. After being dissociated with ACF Enzymatic Dissociation Solution and ACF Enzyme Inhibition Solution (Stemcell Technologies), the iMPCs were grown on regular tissue culture flasks in expansion medium [DMEM/F-12 (Gibco, Grand Island, NY) supplemented with 10% fetal bovine serum (FBS; Gibco), and 1% antibiotics-antimycotics (Gibco)]. iMPCs at passage 4 were used for all experiments.

### Characterization of iMPCs

#### Colony Forming Units (CFU)

One hundred iMPCs were plated on a 100-mm tissue culture dish (Thermo Fisher, Waltham, MA) and cultured at 37°C in 5% humidified CO_2_. After 14 days, the dishes were washed with phosphate-buffered saline (PBS) and stained with 0.5% Crystal Violet (Sigma, St. Louis, MO) in methanol for 5–10 min at room temperature. After extensive washing with PBS, visible colonies in purple were counted.

#### Flow Cytometry Analysis

iMPCs were trypsinized, washed and incubated with the following antibodies: FITC Mouse Anti-Human CD31, CD34, CD45, CD73, and CD90 (BD Biosciences, Franklin Lakes, NJ). Cells were then analyzed by flow cytometry (BD FACS Aria^TM^ II cell sorter; BD Biosciences) to assess the expression of these cell surface epitopes.

### Tri-lineage Differentiation Assays

#### Osteogenesis

iMPCs were seeded at a density of 20,000 cells/cm^2^ into six-well plates and cultured in standard osteogenic medium (OM: high-glucose Dulbecco's modified Eagle medium (DMEM, Gibco) supplemented with 10% FBS, 1% antibiotics-antimycotics, 0.1 μM dexamethasone (Sigma), 10 mM β-glycerophosphate (Sigma), and 50 μg/mL ascorbate 2-phosphate(Sigma) (Tuli et al., 2003). After 21 days, the cultures were fixed with 4% paraformaldehyde and assessed with Alizarin Red staining (Rowley Biochemical, Danvers, MA). Gene expression of typical osteogenic markers, i.e., osteocalcin (*OCN*), alkaline phosphatase (*ALP*) Runt-related transcription factor 2 (*RUNX2*), osteopontin (*OPN*), and bone sialoprotein 2 (*BSP2*), was assay by quantitative real-time polymerase chain reaction (RT-PCR).

#### Chondrogenesis

iMPCs were seeded at 20,000 cells/cm^2^ into six-well plates and cultured in chondrogenic medium [CM: high-glucose DMEM supplemented with 1% antibiotics-antimycotics, 0.1 μM dexamethasone, 40 μg/mL L-proline (Sigma, St. Louis, MO), 10 μg/mL ITS+ (ThermoFisher, Waltham, MA), 50 μg/mL ascorbate 2-phosphate, 10 ng/mL transforming growth factor (TGF)-β3 (Peprotech, Rocky Hill, NJ)] (Tuli et al., 2003). After 21 days, the cultures were fixed with 4% paraformaldehyde and assessed with Alcian Blue staining (EKI, Joliet, IL). Gene expression of typical chondrogenic markers, i.e., SRY-box transcription factor 9 (*SOX9*), collagen type II (*COL2*), and aggrecan (*ACAN*), was analyzed by quantitative RT-PCR.

#### Adipogenesis

iMPCs were seeded at 20,000 cells/cm^2^ into six-well plates and cultured in adipogenic medium (AM: α-MEM (Gibco, Grand Island, NY) supplemented with 10% FBS, 1% antibiotics-antimycotics, 0.45mM 3-isobutyl-1-methylxanthine (Sigma, St. Louis, MO), 0.1 μM dexamethasone, 0.2 mM indomethacin (Sigma, St. Louis, MO), 1 μg/ml ITS+). After being cultured for 21 days, the cells were fixed with 4% paraformaldehyde and assessed with Oil Red staining. Gene expression of typical adipogenic markers, i.e., peroxisome proliferator-activated receptor gamma (*PPAR-*γ), lipoprotein lipase (*LPL*) and adipsin, was analyzed by quantitative RT-PCR.

Cells treated with CM without the supplement of TGF-β3 (Basic Medium, BM) served as the negative control for chondrogenic differentiation. For osteogenesis and adipogenesis, iMPCs grown in the maintenance medium (MM, high-glucose DMEM supplemented with 10% FBS and 1% antibiotic-antimycotic) were used as the control.

### RNA Isolation and Gene Expression Analysis

Samples were lysed in Qiazol Reagent (QIAGEN, Germantown, MD, USA), and RNA was isolated using the RNeasy Plus Universal Mini Kit (QIAGEN, Germantown, MD, USA). Reverse transcription was performed using SuperScript™IV VILO™Master Mix (Invitrogen, Waltham, MA). RT-PCR was performed on an Applied Biosystems RT-PCR system using SYBR Green Reaction Mix (Applied Biosystems, Foster City, CA, USA). Relative gene expression was calculated using the ΔΔCt method, with gene expression levels normalized to that of the housekeeping gene ribosomal protein L13A (*RPL13A*) or glyceraldehyde 3-phosphate dehydrogenase (*GAPDH*). The sequences of primers for each gene are listed in Table S1.

### Synthesis of Methacrylated Gelatin (mGL) and Photoinitiators

mGL and the photoinitiator, lithium phenyl-2,4,6-tri-methylbenzoylphosphinate (LAP), were synthesized as previsouly reported (Lin et al., 2014a). The lyophilized mGL was dissolved in Hanks' Balanced Salt solution (HBSS) (HyClone, Logan, UT) at a concentration of 15% (w/v), with 0.15% LAP added as the photoinitiator.

### Optimization of Chondrogenic and Osteogenic Differentiation of iMPCs

iMPCs pellets were re-suspended in mGL/LAP solution at a density of 20 × 10^6^ cells/mL, transferred into a silicone mold and cured with UV light (395 nm) for 2 min. The final dimensions for cylindrical iMPCs-laden constructs were 5 mm in diameter and 2 mm in height.

In our previous experience, with the standard CM, iMPCs were unable to achieve a comparable level of chondrogenesis as primary MSCs. Bone morphogenetic protein-6 (BMP6) had been identified as a key chondro-inductive growth factor for adipose stem cells (ASCs) (Estes et al., 2006; Diekman et al., 2010). Therefore, 100 ng/ml BMP6 was added to CM and its efficacy in enhancing iMPCs chondrogenesis examined. To optimize osteogenesis, we tested the supplementation of 1α, 25-dihydroxyvitamin D3 (VitD3) (Lou et al., 2017) and an osteogenic protein BMP7 (Luu et al., 2007). We also examined if the withdrawal of dexamethasone (Dex) at a later stage could enhance osteogenesis (Subramaniam et al., 1992; Rimando et al., 2016). As shown in Figure 2A, five different osteogenic medium compositions were compared: Group 1 (Gr 1), MM (negative control); Gr 2, OM; Gr 3, OM + 10 nM VitD3; Gr 4, OM + 10 nM VitD3 + 100 ng/ml BMP-7; Gr 5, OM + 10 nM VitD3 + 100 ng/ml BMP-7, with Dex treatment only during the first 14 days.

All cultures were collected after 28 days of differentiation. The expression levels of chondrogenic marker genes (*SOX9, ACAN*, and *COL2A1*), osteogenic marker genes, *OCN, ALP, OPN*), and *BSP2*, were analyzed by quantitative RT-PCR. The deposition of glycosaminoglycans (GAGs) in the cartilage scaffolds were assessed histologically with Alcian blue staining and quantified by the 1,9-dimethylmethylene blue dye-binding assay (Blyscan, Biocolor, United Kingdom). DNA content was measured using the Picogreen dsDNA assay (Molecular Probes, Tarrytown, NY) for GAGs normalization. Calcium deposition was evaluated by Alizarin Red staining. Expression level of *OCN* and *COL2* in constructs were further examined through immunohistochemistry.

### Fabrication of Osteochondral Tissue Chips

To fabricate OC tissue, iMPCs were suspended in 15% mGL and pipetted into the insert of the microbioreactor (Lin et al., 2014b), followed by photocrosslinking of the mGL hydrogel. Afterwards, the inserts with iMPCs-laden mGL were placed into the bioreactors (Figure 3A). Then, the optimized chondrogenic medium was perfused into the top of the constructs, and the optimized osteogenic medium was supplied through the bottom conduit. The chondrogenic/osteogenic differentiation process lasted for 28 days with the medium flow rate set at 5 μL/min.

To fabricate chondral tissue only constructs (CH), cylindrical scaffolds (*d* = 3 mm; *h* = 2 mm) were first prepared from iMPCs-laden mGL solution in silicone molds. These scaffolds were then placed into the top part of the inserts, with the other half of the insert filled with cell-free mGL. Additional 1.5-min photo-crosslinking was used to fully cure the hydrogel. All bioreactor culture conditions were the same to those used to fabricate OC tissue (Figure 3D).

The OC and CH tissues were characterized by quantitative RT-PCR, histology, as well as immunohistochemistry.

### Immunohistochemistry

All samples were fixed in 10% buffered formalin phosphate, dehydrated and then embedded in paraffin. The blocks were sectioned at 6 μm thickness. For immunohistochemistry (IHC), deparaffinized and rehydrated sections were incubated with primary antibodies against human collagen type II (1:200), osteocalcin and TGF-β3 (1:200) (Abcam, Cambridge, MA) at 4°C overnight, followed by incubation with appropriate secondary antibodies. Immunostaining was carried out using the Vectastain ABC kit and NovaRED peroxidase substrate kit (Vector Labs, Burlingame, CA, USA). Images were acquired with the OLYMPUS CKX41 microscope.

### Creation of Osteoarthritic Phenotype in Osteochondral Tissue Chip

IL-1β (1 ng/ml) was introduced via medium feed to the cartilage side to induce OA-like phenotype (Figure 5A). The phenotype of tissues were assessed using the methods described above.

### Drug Test on Osteoarthritic Osteochondral Tissue Chip

To validate the utility of the iPSC-derived OC tissue chip for drug screening applications, Celecoxib, a COX-2 inhibitor commonly used as a first-line treatment option for OA, was chosen to assess its efficacy. Celecoxib was used at 10 μM in this study (Mastbergen et al., 2005; Su et al., 2014; Cho et al., 2015). Typically, Celecoxib functions systemically, which was simulated in the “SY” group (Figure 6A). In this study, we were also interested in exploring the potential of intraarticular (IA) application of Celecoxib in treating OA, which was simulated in the “IA” group. Normal OC tissues without any treatments served as the control (CL). After 7 days, the therapeutic effects of Celecoxib in both models were evaluated by quantitative RT-PCR and histology.

### Statistical Analysis

Statistical analyses were performed with one-way and two-way analysis of variance (ANOVA) or two-tailed *t*-tests using GraphPad Prism7 (GraphPad Software, San Diego, CA, USA). A *p* < 0.05 *value* was considered statistically significant.

## Results

### iMPCs Displayed MSC-Like Phenotype

As shown in Figure S1, the undifferentiated iPSCs grew in compact colonies with distinct borders and well-defined edges. P4 iMPCs displayed a spindle-like morphology, which was very different from the starting iPSCs. To assess the MSC-like nature of iMPCs, the conventional methods were used, such as CFU assay, tri-lineage differentiation and surface marker profiling.

Similar to MSCs, iMPCs attached to the plastic surface and formed colonies. As shown in Figure S2A, the CFU activities of the two types of cells were comparable. In the tri-lineage differentiation analysis, iMPCs successfully differentiated toward chondrogenic, osteogenic and adipogenic lineages with appropriate stimulation. For example, the expression levels of chondrogenic markers (*SOX9, COL2, ACAN*), osteogenic markers (*RUNX2, ALP, OCN*), and adipogenic markers (*PPAR*γ*2, LPL*, and Adipsin) were all upregulated after induced differentiation. Histological staining further confirmed successful differentiation, indicated by more intense Alcian blue, Alizarin Red, and Oil red staining in cultures treated with chondrogenic, osteogenic, and adipogenic induction media, respectively, compared to those in control medium (Figure S2B).

Flow cytometry results showed that the iMPCs were negative for CD31, CD34, and CD45, and positive for CD73 and CD90 (Figure S2C). Taken together, these findings indicate that iMPCs possess a phenotype similar to human primary MSCs.

### BMP6 Enhanced Chondrogenesis of iMPCs Within mGL Scaffolds

**Figure 1:**
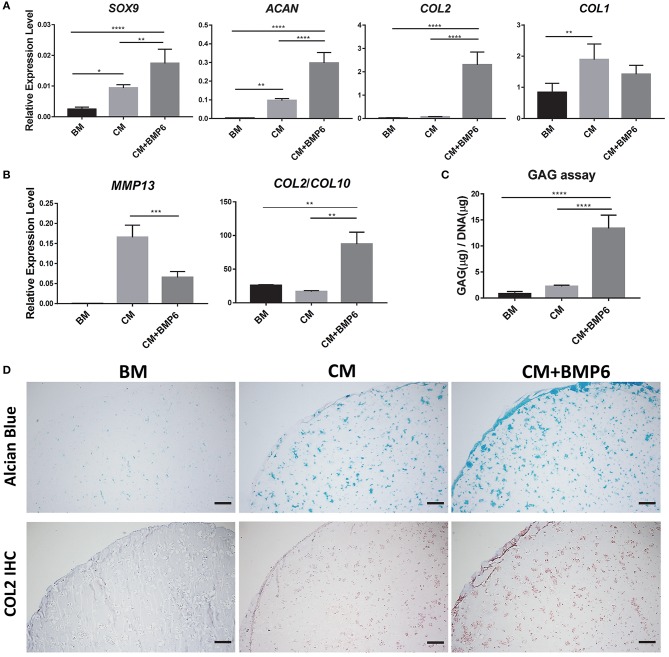
Optimization of chondrogenic medium for iMPCs seeded within mGL scaffolds. **(A)** Expression of chondrogenic marker and *COL1* in constructs treated with BM, CM, or CM+BMP6. All data are normalized to the endogenous control gene, RPL13A. Values are presented as mean ± SD (*N* = 4 per group; *p < 0.05; **p < 0.01; ****p < 0.0001). **(B)** Expression of hypertrophy marker genes in BM, CM, and CM+BMP6 groups. All data are normalized to RPL13A. *N* = 4 per group; **p < 0.01; ***p < 0.001. **(C)** GAG content in the constructs from BM, CM, and CM+BMP6 groups. *N* = 4 per group; ****p < 0.0001. **(D)** Alcian Blue staining (top) and collagen type II IHC (bottom) for the constructs from different groups. Blue (top panel) and brown (bottom panel) indicate positive staining. Bar = 200 μm.

Conventional TGF-β3 containing CM showed limited efficacy in promoting iMPCs chondrogenesis (Figure S2B). This necessitated the development of a more optimal chondrogenic medium for iMPCs. As shown in Figure 1A, the addition of BMP6 into CM significantly enhanced the expression of chondrogenic genes, *SOX9, COL2*, and *ACAN*. Besides, we saw the significant increase of collagen type 1 (*COL1*) in CM group, but not in CM+BMP6, suggesting that the optimal chondrogenic medium (CM+BMP6) has the capability of suppressing fibrocartilage formation. Interestingly, *MMP13* was downregulated in the CM + BMP6 group (Figure 1B). The ratio of *COL2/COL10* expression level was also enhanced, suggesting reduced hypertrophy phenotype with the treatment of BMP6. To further assess the outcome of chondrogenesis, GAG/dsDNA was measured (Figure 1C). The cells in constructs containing CM + BMP6 produced about 5 times more GAG than those in the CM group. The Alcian Blue staining and collagen type II immunohistochemistry further confirmed more deposition of cartilage matrix in the CM+BMP6 (Figure 1D). Based on these results, CM + 100 ng/ml BMP6 was used as the chondrogenic medium for iMPCS in subsequent experiments.

### iMPCs Displayed a Robust Osteogenic Capacity in mGL

**Figure 2:**
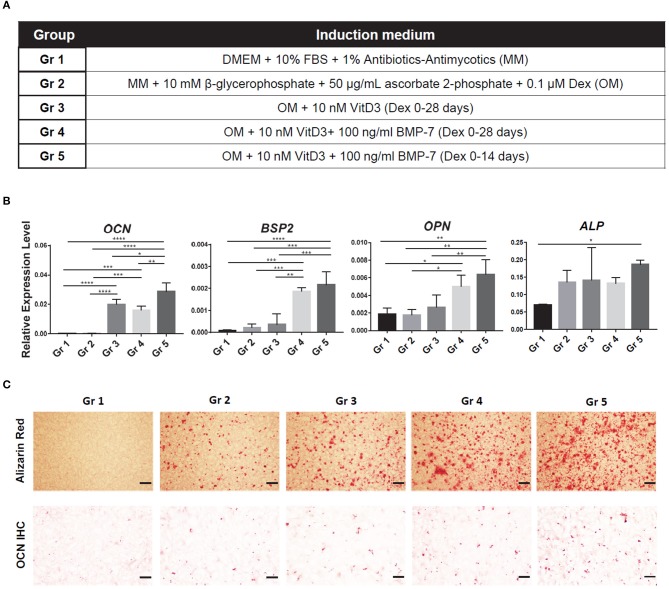
Optimization of osteogenic medium for iMPCs seeded within mGL scaffolds. **(A)** Five different media are listed as Group (Gr) 1-5. The components of each medium group are listed in Table. **(B)** Expression of osteogenic markers in constructs treated with each medium group. All data are normalized to RPL13A. *N* = 4 per group; *p < 0.05; **p < 0.01; ***p < 0.001; ****p < 0.0001. **(C)** Alizarin Red staining (top) and osteocalcin IHC (bottom) for the constructs from each group. Red (top panel) and brown (bottom panel) indicate positive staining. Bar = 200 μm.

To further enhance the osteogenesis of iMPCs, five types of medium were tested (Figure 2A). The expression levels of representative osteogenic marker genes were examined (Figure 2B). The application of VitD3 significantly enhanced the expression of *OCN*, while BMP7 resulted in a higher expression of *BSP2* and *OPN*. The reduction of Dex treatment time from 28 to 14 days did not affect the expression of these tested genes, but enhanced mineralization and deposition of OCN (Figure 2C). These results indicated that OM, with the supplement of VitD3 or BMP-7, resulted in enhanced osteogenesis of iMPCs. Also, a shorter treatment time of Dex might be beneficial to bone matrix formation. The medium from Group 5 was used in all the following experiments.

### Generation of Biphasic Osteochondral Tissue From iMPCs

**Figure 3:**
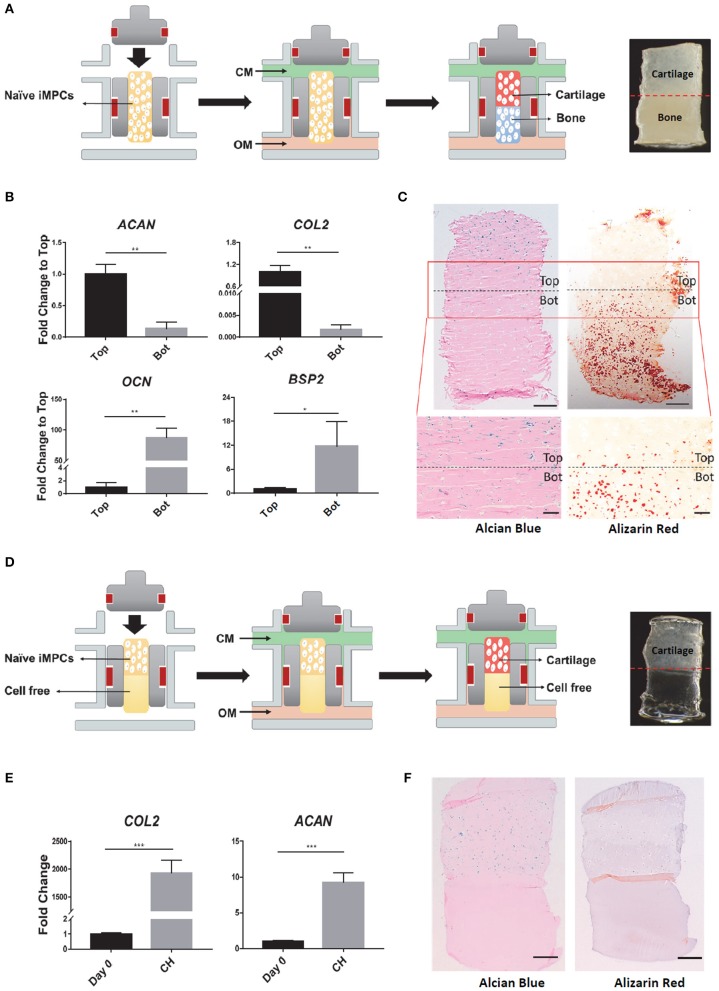
Fabrication of osteochondral (OC) or chondral (CH) tissue in the dual-flow bioreactor. **(A)** Schematic of generating OC tissue. After naïve iMPCs were encapsulated into mGL and placed into bioreactor, optimized chondrogenic, and osteogenic media were perfused through the top and bottom flow, respectively, to Induce the formation of the bi-phasic OC tissue, with cartilage in the top and bone in the bottom. **(B,C)** Characterization of the engineered OC construct. **(B)** Expression levels of chondrogenic (*AGAN, COL2*) and osteogenic (*OCN, BSP2*) marker genes in the top and bottom (Bot) parts of OC construct. Gene expression levels in the top component are normalized as 1. *N* = 3 per group; *p < 0.05; **p < 0.01. **(C)** Alcian Blue and Alizarin red staining for OC construct. Bar, top panel = 500 μm; Bar, bottom panel = 200 μm. **(D)** Schematic of generating CH tissue. All the culture conditions were the same to those used in generating OC, except that no cells were encapsulated in the bottom part. **(E,F)** Characterization of the engineered CH construct. **(E)** Expression levels of *ACAN, COL2* in the constructs after 28 days differentiation. Expression levels of Day 0 are set as 1. *N* = 3 per group; ***p < 0.001. **(F)** Alcian Blue and Alizarin red staining for CH construct. Bar = 500 μm.

The naïve iMPCs were encapsulated into mGL and placed into a dual-flow bioreactor developed in our lab (Figure 3A). The optimized chondrogenic and osteogenic media described earlier were perfused through the top and bottom flows, respectively, into the bioreactor. After 28 days of differentiation, the tissue phenotype of the biphasic construct was characterized. Quantitative RT-PCR results showed that the top “chondral” part of tissues exhibited significantly higher expression levels of *COL2* and *ACAN* than the bottom. In contrast, the cells from the “osteo” bottom showed higher expression levels of *OCN* and *BSP2* (Figure 3B). The formation of biphasic OC tissues were further examined histologically in terms of deposition of tissue specific matrix. As shown in Figure 3C, only the top part of the construct was positive for Alcian Blue staining, while robust Alizarin Red staining was only observed in the bottom part. Taken together, these results suggested the robust formation of an osteochondral tissue within the bioreactor, in which a cartilage-like tissue, osteochondral-cartilage (OC-C), was formed in the top part and bone-like tissue, osteochondral-bone (OC-B), in the bottom.

Monophasic chondral tissue (CH) was also engineered after 28 days of bioreactor culture (Figure 3D). Quantitative RT-PCR results showed the enhanced gene expression of *COL2* and *ACAN*, compared to undifferentiated tissue at Day 0 (Figure 3E). Positive Alcian Blue staining was only observed in the top part. Alizarin Red staining was negative in the entire construct (Figure 3F), indicating the formation of a monophasic chondral tissue.

### Chondrosupportive Activity of Bone Component in the Engineered Osteochondral Tissue

**Figure 4:**
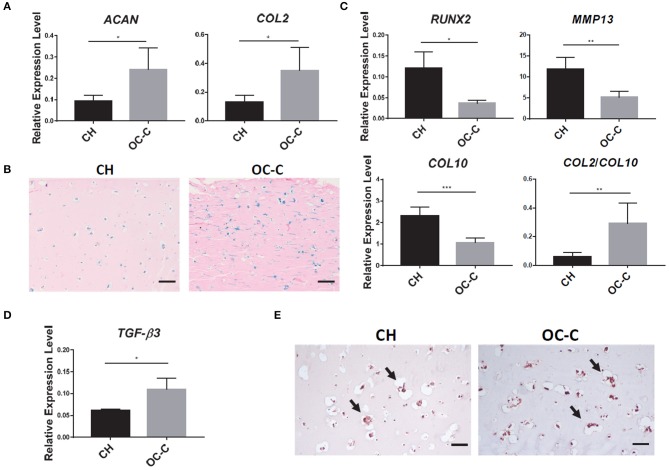
Beneficial effect of bone component in promoting chondrogenesis in OC. **(A)** Cartilage in OC (OC-C) showed higher expression of *ACAN* and *COL2* than CH. All data are normalized to RPL13A. *N* = 4 per group; *p < 0.05. **(B)** OC-C showed more Alcian blue staining, indicating higher GAG deposition (Bar = 200 μm). **(C)** OC-C displayed lower level of hypertrophy than CH alone, indicated by lower expression of *RUNX2, MMP13, COL10*, and higher *COL2/COL10* ratio. All data are normalized to RPL13A. *N* = 4 per group; *p < 0.05; **p < 0.01; ***p < 0.001. **(D)** *TGF-*β*3* expression level in OC-C and CH. Data are normalized to RPL13A. *N* = 4 per group; *p < 0.05. **(E)** IHC for TGF-β3. Bar = 100 μm.

To explore the influence of subchondral bone on the health of cartilage under normal physiology conditions, we compared the phenotype of OC-C with CH in terms of chondrogenic gene expression and histology. As shown in Figures 4A,B, OC-C displayed significantly higher expression of *COL2* and *ACAN* as well as more GAG deposition than that in CH. Interestingly, compared to single CH tissue, the hypertrophy marker genes, including *COL10, MMP13*, and *RUNX2*, were also significantly suppressed in the OC-C (Figure 4C). To gain insight into the underlying mechanism of the enhanced chondrogenesis in OC-C, we analyzed the expression level of TGF-β3, a robust chondro-inductive factor. The results in Figures 4D,E showed a significantly higher level of *TGF-*β*3* expression in OC-C, as well as more intense pericellular TGF-β3 immunostaining, which likely contributed to the enhanced chondrogenesis seen in OC-C.

### Development of OA Model Using Osteochondral Tissue Chip

**Figure 5:**
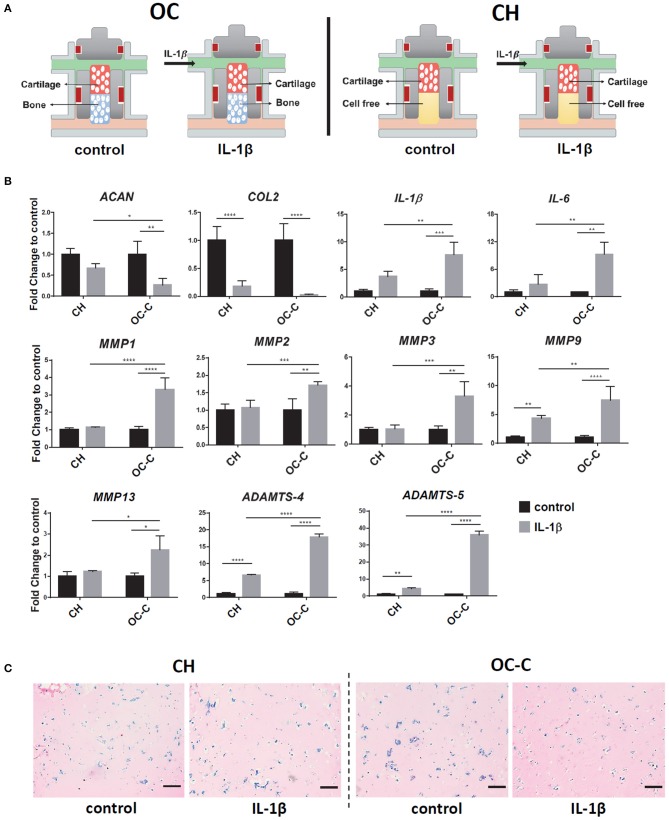
Creation of OA model in iMPCs-derived tissue chip. **(A)** Schematic of the process. After 28 days of differentiation and the formation of OC or CH, 1 ng/ml IL-1β was introduced into the top flow and perfused only onto the cartilage component. **(B)** Expression levels of anabolic factors (*ACAN, COL2*), proinflammatory cytokines (*IL-1*β*, IL-6*), and catabolic factors in OC or CH, with or without the treatment of IL-1β. All data are normalized to RPL13A. *N* = 4 per group. *p < 0.05; **p < 0.01; ***p< 0.001; ****p< 0.0001. **(C)** Alcian Blue staining of cartilage from OC or CH samples, with or without the treatment of IL-1β. Bar = 200 μm.

We next used the OC tissue chip to create an OA model, by challenging the cartilage tissue with IL-1β (Figure 5A). As shown in Figure 5B, treatment of IL-1β decreased the expression of *COL2* and *ACAN* in both OC-C and CH constructs.

Interestingly, the IL-1β effect on *COL2* expression was significantly stronger in OC-C (20-fold decrease), compared to CH (5-fold decrease). In addition, Alcian blue staining showed less GAG in IL-1 β treated OC-C than CH (Figure 5C). To explore the underlying mechanism of enhanced degeneration in OC-C, we examined the expression levels of cartilage degenerating enzymes and proinflammatory cytokines. Results in Figure 5B showed that IL-1β treated OC-C exhibited significantly greater fold change of *MMP1, 2, 3, 9, 13, ADAMTS4, 5, IL-1*β, and *IL-6* than CH tissue, when the expression levels of these genes were normalized to their own control (without the treatment of IL-1β). These findings indicated that the presence of OC-B could aggravate the degeneration of OC-C under the treatment of IL-1β.

### Therapeutic Effect of Celecoxib in OA Chip Model

**Figure 6:**
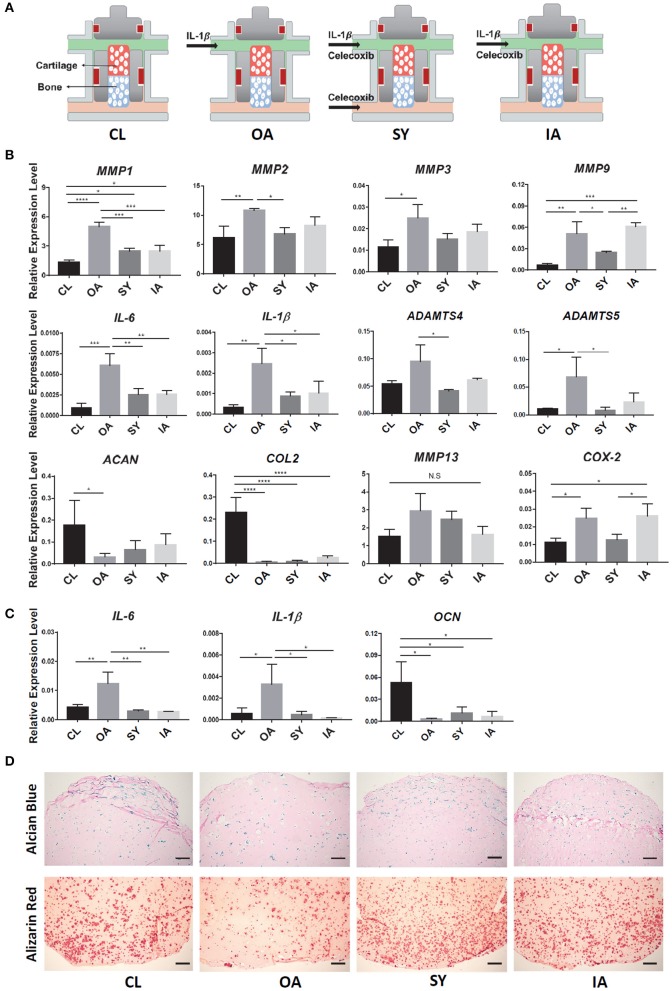
Testing disease-modifying effects of Celecoxib in iPSC-derived OC chip-based OA model. **(A)** Schematic of treatments. After the formation of OC tissue, IL-1β was used to generate “OA” model (OA group). Administration of Celecoxib to both cartilage and bone components of OC chip simulated systemic treatment (SY group), while administration tocartilage alone simulated intraarticular treatment (IA group). Normal OC tissues without any treatment served as the control (CL). **(B)** Expression levels of catabolic genes in the cartilage part of OC and CH constructs. All data are normalized to RPL13A. *N* = 4 per group; *p < 0.05; **p < 0.01; ***p < 0.001; ****p< 0.0001. **(C)** Expression levels of *IL-1*β*, IL-6*, and *OCN* in the bone side of OC tissue. All data are normalized to RPL13A. *N* = 4 per group. *p < 0.05; **p < 0.01. **(D)** Alcian Blue (top) and Alizarin Red (bottom) staining of samples from OC constructs with different treatments. Bar = 200 μm.

To test the utility of iPSC-derived OC tissue chip in drug screening, Celecoxib, a selective cyclooxygenase 2 (COX-2) inhibitor commonly used to treat OA, was administered to the system. Celecoxib was administered at 10 μM concentration and showed no noticeable adverse effect on the health of normal chondral tissue (Figure S3). Administration of Celecoxib to both OC-C and OC-B were used to simulate the typically systemic action of Celecoxib, represented by the “SY” group (Figure 6A). In addition, we also examined the potential effect of intra-articular (IA) application of Celecoxib in treating OA, by delivery only to OC-C, namely the “IA” group. Normal OC tissues without any treatments served as the control (CL).

After 7 days of Celecoxib treatment, the expression levels of representative catabolic factors, *MMP1, 2, 9, ADAMTS-4, 5*, and inflammatory factors, *IL-1*β, *IL-6* and *COX2*, were significantly suppressed in the IL-1β-treated OA chips (Figure 6B). A trend of downregulation was also observed for *MMP3* and *MMP13*, although there was no significant difference. The expression levels of *ACAN* and *COL2* were also partly rescued after Celecoxib treatment, suggesting the potential disease-modifying effect of Celecoxib. In particular, compared to the “IA” group, better therapeutic effects of Celecoxib were seen in the “SY” group, manifested by significantly lower expression of *MMP9* and *COX-2*.

We also examined the influence of Celecoxib on the health of OC-B. Celecoxib downregulated the expression of *IL-6* and *IL-1*β, and partially rescued *OCN* expression in the bone tissue (Figure 6C), suggesting its osteoprotective effect. Finally, the beneficial effect of Celecoxib on osteoarthritic tissues was further confirmed by histology, showing that both the “IA” and “SY” groups retained more proteoglycan and calcium deposition than the “OA” group (Figure 6D). Moreover, similar to the effect seen in the chondral component of the OC chip, Celecoxib treatment of both tissue components, i.e., the “SY” group, yielded higher staining than the “IA” group, indicating a better osteoprotective effect.

## Discussion

We have successfully developed an iPSC-derived tissue chip to model the OC unit in the human articular joint. We have investigated its physiological relevance, created an OA-like phenotype, and replicated in part the beneficial effect of Celecoxib in modifying OA. To the best of our knowledge, this is the first report on the fabrication of microphysiological OC tissue from iPSCs within a customized bioreactor.

Organ-on-a-chip technology is a new technique that has been gaining increasing interest in recent years. Compared with traditional 2D and single-cell culture models, organ-on-a-chip technology offers possibilities to investigate the cellular behavior and intercellular interactions in a context that simulates human physiology and disease pathology (Bhatia and Ingber, 2014). Both tissue-derived differentiated cells and stem cells have been used as the starting cellular components of organ/tissue chips. Compared to MSCs or other terminally differentiated cell, iPSCs possess pluripotent differentiation potential and represent a potentially infinite cell source, thus allowing a better control of the inherent variability of tissue chips. Of relevance to the construction of OC chips, to date, a variety of methods for *in vitro* chondrogenesis and osteogenesis of iPSCs have been reported, but there is no generally accepted standard protocol. In this study, we first induced iPSCs into an intermediate phenotype of mesenchymal progenitors, termed iMPCs, and then differentiated the cells into chondrocytes and osteoblasts. This strategy was also reported in other studies (Nejadnik et al., 2015; Diederichs et al., 2016; Jeon et al., 2016). We then used established criteria for MSCs to characterize iMPCs. Specifically, MSC-like cells need to possess the characteristics of adherence to plastic, colony formation capacity, multipotent differentiation potential and specific surface antigen expression (Dominici et al., 2006). Our results confirmed the MSC-like characteristics of the iMPCs.

The iMPCs were then subsequently induced into chondrogenic and osteogenic lineages within a photocrosslinked mGL scaffold. Since the iMPCs were generally less responsive to traditional MSC differentiation protocols, particularly chondrogenesis (Diederichs and Tuan, 2014), optimization of chondrogenic and osteogenic media is needed to generate optimal cartilage and bone constructs. Bone morphogenetic proteins (BMPs) are multi-functional growth factors that have unique properties as inducers for the differentiation of multipotent mesenchymal progenitors into chondrocytes and osteoblasts. BMP6 was reported to promote the chondrogenesis of MSCs and ASCs (Sekiya et al., 2001; Estes et al., 2006; Diekman et al., 2010), but its potential on iMPCs has not been examined. Our findings reveal the potent chondrogenic effect of BMP6 on iMPCs, on the basis of significant improvement in chondrogenic gene expression, as well as GAG production, shown quantitatively and histologically. In addition, BMP6 is also observed to exhibit anti-hypertrophic effect. BMP6-mediated promotion of chondrogenesis of adipose-derived MSCs has been reported as being mediated via the enhanced expression of TGFβ-receptor-I (Hennig et al., 2007). The underlying mechanism of BMP6-enhanced iMPC chondrogenesis deserves further investigation in the future.

Currently, Dex is a common supplement in osteogenic and chondrogenic media for MSCs (Tuli et al., 2003; Derfoul et al., 2006). However, the optimal duration of Dex treatment is quite controversial. On one hand, Dex increased the expression of *RUNX2* and its transcriptional targets, *ALP* and *OSX*. On the other hand, Dex significantly downregulated the expression of late osteogenic markers, *COL1A1, OPN, OCN*, and *BSP2* (Subramaniam et al., 1992; Langenbach and Handschel, 2013; Rimando et al., 2016). Therefore, we hypothesized that extended Dex treatment is likely to inhibit osteogenesis. It is noteworthy that BMP7, a strong osteoinductive factor (Miyazono et al., 2005), and VitD3, a known promoter of bone formation *in vivo* and *in vitro* (Rimando et al., 2016), have not been commonly included in the osteogenic medium for MSCs, particularly BMP7. Our previous studies have shown the beneficial effect of VitD3 on MSC osteogenesis (Jackson et al., 2009; Djouad et al., 2010). In this study, we have incorporated these features in optimizing the osteogenic medium. The results in Figure 2 clearly show that the inclusion of BMP7 and VitD3 in OM and reduction of Dex treatment time to 14 days, resulted in the highest level of osteogenic differentiation of iMPCs.

In order to form biphasic bone and cartilage from uniform stem cell-laden constructs, we need to completely separate the osteogenic and chondrogenic medium. The porous scaffolds may cause free exchange. Therefore, the hydrogel is the best option because of its ability to fully fill the insert and seal the gap between scaffold and insert wall, thus preventing direct medium mixing. In particular, we have successfully utilized photocrosslinkable gelatin in create bone and cartilage, which thus was employed here as the scaffold to support the formation of osteochondral tissue (Lin et al., 2014a,b, 2019). Through the application of optimized chondro- and osteo-inductive conditions, we have successfully engineered a biphasic OC tissue as well as a monophasic cartilage tissue in the customized bioreactor. While hyaline cartilage and bone have distinct structural and compositional differences, their juxtaposition in the osteochondral junction strongly suggests a functional relationship between the two tissue components. In addition to the distribution of the biomechanical force loaded to the cartilage, the subchondral bone is functionally essential in maintaining articular cartilage viability (Amin et al., 2009). Specifically, the non-mineralized structural space in the osteochondral junction allows the transportation of growth factors and signaling molecules (Arkill and Winlove, 2008; Pan et al., 2009), thus enabling active biochemical crosstalk between cartilage and subchondral bone under physiological conditions. Thus, subchondral bone is of great importance to the healthy development and maintenance of the overlying cartilage. We demonstrated here the essential role of subchondral bone in promoting and maintaining cartilage phenotype and inhibiting cartilage hypertrophy in the healthy condition. These chondro-promoting effects of bone tissue were probably realized through the upregulation of TGF-β production in cartilage, since members of the TGF-β superfamily are involved in cartilage formation, maturation, and maintenance (Wang et al., 2014; van der Kraan, 2017). As expected, we found enhanced TGF-β3 production in the chondral tissue when maintained in the presence of bone tissue in the context of a biphasic OC construct. This finding supports previous studies that showed the positive effects of TGF-β in the initial stages of chondrocyte differentiation and proteoglycan synthesis, as well as terminal hypertrophy (Morales et al., 1991; Ballock et al., 1993; Yang et al., 2001; Zhang et al., 2004; Mueller and Tuan, 2008; Mueller et al., 2010; Shintani et al., 2013). Aside from TGF-β, morphogenic signals produced by bone could also promote chondrogenesis. It is reported that inhibition of BMP signaling could suppress the formation of cartilage (Kawakami et al., 1996) and knockout of BMP receptors in mice affected precartilaginous condensations, resulting in inhibition of chondrocyte formation and defective maturation of chondrocytes (Yoon et al., 2005, 2006).

To model OA conditions in the OC tissue chip, IL-1β (1 ng/ml) was applied to the chondral tissue (OC-C) to initiate an inflammatory response. Cartilage degeneration is usually mediated by the action of the enzymes, MMPs and ADAMTSs, which degrade collagen and aggrecan of the cartilage extracellular matrix (Murphy and Lee, 2005). Studies have shown that human OA cartilage exhibits enhanced production of MMP1, 2, 3, 8, 9, 13, TNF-α, IL-1β, IL-6, and ADAMTS-4, 5 (Tetlow et al., 2001; Malfait et al., 2002; Qu et al., 2015). In the *in vitro* OA model reported here, we observed similar changes, thus validating the physiological relevance of the human cell-based OC chip OA model.

With the progression of OA, permeability between cartilage and bone is increased, as validated in previous studies by testing hydraulic conductance in normal and OA osteochondral samples (Hwang et al., 2008; Stender et al., 2017). Several *in vitro* studies using co-cultures of chondrocytes with sclerotic or overloaded osteoblasts have also provided evidence for direct catabolic effect of the subchondral bone on cartilage degradation (Sanchez et al., 2005, 2015; Priam et al., 2013). In fact, subchondral bone abnormality can eventually lead to the degeneration of overlying articular cartilage (Zhen et al., 2013; Xu et al., 2015), and this relationship has been suggested in OA pathogenesis (Cope et al., 2019). Our findings in this study show that compared to the cartilage only group (CH), IL1β-challenged OC-C exhibited significantly higher fold increase of inflammatory and catabolic gene expression levels, suggesting the aggravating role of bone in inflammation-mediated cartilage degeneration.

Currently, there are no FDA-approved DMOADs. Non-steroidal anti-inflammatory drugs (NSAIDs) have been widely used to relieve pain and inflammation, and improve the mobility of OA patients (Sarzi-Puttini et al., 2005). In addition to their anti-inflammatory effects, NSAIDs may also prevent cartilage matrix degradation by inhibiting proteolytic enzymes, including ADAMTS and MMPs (Zweers et al., 2011; Nakata et al., 2018), and therefore have potential as DMOADs. In this study, we tested Celecoxib in the OC tissue chip-based OA model. Celecoxib, a COX-2 inhibitor that has demonstrated significantly improved gastrointestinal safety and tolerability with no less effectiveness than other NSAIDs for relief of OA symptoms (Deeks et al., 2002), was chosen to test the capability of the OC chip as a DMOAD screening platform. Specifically, we simulate both systemic (SY) and intra-articular (IA) routes of drug administration by introducing Celecoxib to: (1) both the bone and cartilage components of the biphasic model, or (2) only the cartilage component in the biphasic OC model, respectively. Celecoxib treatment significantly reduced inflammation and catabolism in chondral tissues. We observed the downregulation of *MMP1*, 2, *3, 9*, 13, *IL-6, IL-1*β, and *ADAMTS4* and *5* after Celecoxib treatment. The expression levels of *COL2* and *ACAN* were also enhanced, although no statistical differences were observed. The disease modifying activity of Celecoxib found in our study is comparable to the chondroprotective effect of Celecoxib reported in previous studies, in which Celecoxib not only stimulated proteoglycan synthesis and retention of newly formed proteoglycans in OA cartilage explants (El Hajjaji et al., 2003; Mastbergen et al., 2005, 2006), but also reduced the expression of *ADAMTS-5, IL-1*β, *IL-6*, and *MMP1, 3, 9* in OA cartilage or chondrocytes (Sanchez et al., 2002; Alvarez-Soria et al., 2008; Attur et al., 2008; Tsutsumi et al., 2008; Su et al., 2014). In another study, after Celecoxib treatment *in vivo* for 4 weeks, human OA cartilage tissue showed significant beneficial effects on proteoglycan synthesis, release, and content (de Boer et al., 2009). Intra-articular injection of Celecoxib in a rabbit OA model was also reported to mitigate pathological changes and significant suppression of IL-1β, TNF-α, and MMP-3 (Jiang et al., 2010). Although opposite results about the effects of Celecoxib on chondrocytes have also been reported (Nakamura et al., 2007; Raynauld et al., 2010), our results using the OC tissue chip support the OA-modifying effects of Celecoxib. It is noteworthy that compared to the “IA” group, better therapeutic effects of Celecoxib were seen in the “SY” group, manifested by significantly lower expression of *MMP9* and *COX2*. Moreover, Celecoxib also exhibited osteoprotective effect, downregulating the expression of *IL-6* and *IL-1*β, and partially rescued *OCN* expression in the OC-B component in the OC OA model.

While the engineered iPSC-based OC tissue chip represents a highly relevant microphysiological model of the articular joint, there are some obvious limitations. For example, a tidemark-like structure, an important component in the native osteochondral junction, is not observed in the tissue chip. Although we observed enhanced TGF-β3 expression in the cartilage co-cultured with bone (Figure 4D), the exact factor(s) from bone that cause such enhancement has not been identified. In the next step, we will try to use a proteomics analyzing tool such as mass spectrometry, to explore potential signaling factors. In addition, only chondrocyte- and osteoblast-like cells are present in the chip. Given the fact that OA is a disease of the whole joint and affects all the cells/tissues in the joint, other tissue elements, such as the synovium and infrapatellar fat pad, need be integrated in future studies. Lastly, additional drugs that have shown varying therapeutic effects on the OA joint need to be tested in order to validate the suitability of the iPSC-based OC tissue chip as a drug screening platform to predict therapeutic efficacy and potential toxicity.

## Conclusion

We have successfully created an *in vitro* OC chip model from iPSCs, which is capable of displaying an OA-like phenotype after the treatment with the pro-inflammatory cytokine, IL1-β. Our findings have demonstrated the physiological crosstalk between bone and cartilage, specifically that bone promotes chondral phenotype in cartilage under healthy conditions, but augments cartilage degradation under degenerative, OA-like conditions. The anti-inflammatory and anti-degenerative effects of the NSAID, Celecoxib, were replicated in the OC chip-based OA model, suggesting its potential application as a drug screening platform for candidate DMOADs.

## Data Availability Statement

The raw datasets generated for this study can be found in the GEO, accession numbers listed in Supplementary Material.

## Author Contributions

ZLin conducted all experiments and data analyses and wrote the manuscript. HL, RT, and PA designed all experimental procedures, supervised the project, and revised the manuscript. ZLi, EL, XL, CD, HS, TH, BO'D, SG, and BB contributed to the implementation of the research and/or to the analysis of the results. All authors edited the manuscript.

### Conflict of Interest

The authors declare that the research was conducted in the absence of any commercial or financial relationships that could be construed as a potential conflict of interest.

## References

[B1] Alvarez-Soria M. A. Herrero-Beaumont G. Moreno-Rubio J. Calvo E. Santillana J. Egido J.. (2008). Long-term NSAID treatment directly decreases COX-2 and mPGES-1 production in the articular cartilage of patients with osteoarthritis. Osteoarthr. Cartil. 16, 1484–1493. 10.1016/j.joca.2008.04.022 18547825

[B2] Amin A. K. Huntley J. S. Simpson A. H. Hall A. C. (2009). Chondrocyte survival in articular cartilage: the influence of subchondral bone in a bovine model. J. Bone Joint Surg. Br. 91, 691–699. 10.1302/0301-620X.91B5.21544 19407309

[B3] Arkill K. P. Winlove C. P. (2008). Solute transport in the deep and calcified zones of articular cartilage. Osteoarthr. Cartil. 16, 708–714. 10.1016/j.joca.2007.10.001 18023368

[B4] Attur M. Al-Mussawir H. E. Patel J. Kitay A. Dave M. Palmer G.. (2008). Prostaglandin E2 exerts catabolic effects in osteoarthritis cartilage: evidence for signaling via the EP4 receptor. J. Immunol. 181, 5082–5088. 10.4049/jimmunol.181.7.5082 18802112

[B5] Ballock R. T. Heydemann A. Wakefield L. M. Flanders K. C. Roberts A. B. Sporn M. B. (1993). TGF-beta 1 prevents hypertrophy of epiphyseal chondrocytes: regulation of gene expression for cartilage matrix proteins and metalloproteases. Dev. Biol. 158, 414–429. 10.1006/dbio.1993.1200 8344460

[B6] Bhatia D. Bejarano T. Novo M. (2013). Current interventions in the management of knee osteoarthritis. J. Pharm. Bioallied Sci. 5, 30–38. 10.4103/0975-7406.106561 23559821 PMC3612336

[B7] Bhatia S. N. Ingber D. E. (2014). Microfluidic organs-on-chips. Nat. Biotechnol. 32, 760–772. 10.1038/nbt.2989 25093883

[B8] Cho H. Walker A. Williams J. Hasty K. A. (2015). Study of osteoarthritis treatment with anti-inflammatory drugs: cyclooxygenase-2 inhibitor and steroids. Biomed Res. Int. 2015:595273. 10.1155/2015/595273 26000299 PMC4427003

[B9] Cope P. J. Ourradi K. Li Y. Sharif M. (2019). Models of osteoarthritis: the good, the bad and the promising. Osteoarthritis Cartilage 27, 230–239. 10.1016/j.joca.2018.09.016 30391394 PMC6350005

[B10] de Boer T. N. Huisman A. M. Polak A. A. Niehoff A. G. van Rinsum A. C. Saris D.. (2009). The chondroprotective effect of selective COX-2 inhibition in osteoarthritis: *ex vivo* evaluation of human cartilage tissue after *in vivo* treatment. Osteoarthr. Cartil. 17, 482–488. 10.1016/j.joca.2008.09.002 18926729

[B11] Deeks J. J. Smith L. A. Bradley M. D. (2002). Efficacy, tolerability, and upper gastrointestinal safety of celecoxib for treatment of osteoarthritis and rheumatoid arthritis: systematic review of randomised controlled trials. BMJ 325:619. 10.1136/bmj.325.7365.619 12242171 PMC126301

[B12] Derfoul A. Perkins G. L. Hall D. J. Tuan R. S. (2006). Glucocorticoids promote chondrogenic differentiation of adult human mesenchymal stem cells by enhancing expression of cartilage extracellular matrix genes. Stem Cells 24, 1487–1495. 10.1634/stemcells.2005-0415 16469821

[B13] Dibonaventura M. Gupta S. McDonald M. Sadosky A. (2011). Evaluating the health and economic impact of osteoarthritis pain in the workforce: results from the National Health and Wellness Survey. BMC Musculoskelet. Disord. 12:83. 10.1186/1471-2474-12-83 21527024 PMC3110556

[B14] Diederichs S. Gabler J. Autenrieth J. Kynast K. L. Merle C. Walles H.. (2016). Differential regulation of SOX9 protein during chondrogenesis of induced pluripotent stem cells versus mesenchymal stromal cells: a shortcoming for cartilage formation. Stem Cells Dev. 25, 598–609. 10.1089/scd.2015.0312 26906619

[B15] Diederichs S. Tuan R. S. (2014). Functional comparison of human-induced pluripotent stem cell-derived mesenchymal cells and bone marrow-derived mesenchymal stromal cells from the same donor. Stem Cells Dev. 23, 1594–1610. 10.1089/scd.2013.0477 24625206 PMC4086513

[B16] Diekman B. O. Estes B. T. Guilak F. (2010). The effects of BMP6 overexpression on adipose stem cell chondrogenesis: interactions with dexamethasone and exogenous growth factors. J. Biomed. Mater. Res. A 93, 994–1003. 10.1002/jbm.a.32589 19722282 PMC3616877

[B17] Djouad F. Jackson W. M. Bobick B. E. Janjanin S. Song Y. Huang G. T.. (2010). Activin A expression regulates multipotency of mesenchymal progenitor cells. Stem Cell Res. Ther. 1:11. 10.1186/scrt11 20637060 PMC2905087

[B18] Dominici M. Le Blanc K. Mueller I. Slaper-Cortenbach I. Marini F. Krause D.. (2006). Minimal criteria for defining multipotent mesenchymal stromal cells. the international society for cellular therapy position statement. Cytotherapy 8, 315–317. 10.1080/14653240600855905 16923606

[B19] El Hajjaji H. Marcelis A. Devogelaer J. P. Manicourt D. H. (2003). Celecoxib has a positive effect on the overall metabolism of hyaluronan and proteoglycans in human osteoarthritic cartilage. J. Rheumatol. 30, 2444–2451. 14677191

[B20] Estes B. T. Wu A. W. Guilak F. (2006). Potent induction of chondrocytic differentiation of human adipose-derived adult stem cells by bone morphogenetic protein 6. Arthritis Rheum. 54, 1222–1232. 10.1002/art.21779 16572454

[B21] Findlay D. M. Kuliwaba J. S. (2016). Bone-cartilage crosstalk: a conversation for understanding osteoarthritis. Bone Res. 4:16028. 10.1038/boneres.2016.28 27672480 PMC5028726

[B22] Hennig T. Lorenz H. Thiel A. Goetzke K. Dickhut A. Geiger F.. (2007). Reduced chondrogenic potential of adipose tissue derived stromal cells correlates with an altered TGFbeta receptor and BMP profile and is overcome by BMP-6. J. Cell. Physiol. 211, 682–691. 10.1002/jcp.20977 17238135

[B23] Hwang J. Bae W. C. Shieu W. Lewis C. W. Bugbee W. D. Sah R. L. (2008). Increased hydraulic conductance of human articular cartilage and subchondral bone plate with progression of osteoarthritis. Arthritis Rheum. 58, 3831–3842. 10.1002/art.24069 19035476 PMC2603273

[B24] Jackson W. M. Aragon A. B. Bulken-Hoover J. D. Nesti L. J. Tuan R. S. (2009). Putative heterotopic ossification progenitor cells derived from traumatized muscle. J. Orthop. Res. 27, 1645–1651. 10.1002/jor.20924 19517576 PMC3014572

[B25] Jeon O. H. Panicker L. M. Lu Q. Chae J. J. Feldman R. A. Elisseeff J. H. (2016). Human iPSC-derived osteoblasts and osteoclasts together promote bone regeneration in 3D biomaterials. Sci. Rep. 6:26761. 10.1038/srep26761 27225733 PMC4881234

[B26] Jiang D. Zou J. Huang L. Shi Q. Zhu X. Wang G.. (2010). Efficacy of intra-articular injection of celecoxib in a rabbit model of osteoarthritis. Int. J. Mol. Sci. 11, 4106–4113. 10.3390/ijms11104106 21152323 PMC2996802

[B27] Kawakami Y. Ishikawa T. Shimabara M. Tanda N. Enomoto-Iwamoto M. Iwamoto M.. (1996). BMP signaling during bone pattern determination in the developing limb. Development 122, 3557–3566. 8951071 10.1242/dev.122.11.3557

[B28] Langenbach F. Handschel J. (2013). Effects of dexamethasone, ascorbic acid and β-glycerophosphate on the osteogenic differentiation of stem cells *in vitro*. Stem Cell Res. Ther. 4, 117. 10.1186/scrt328 24073831 PMC3854789

[B29] Le B. Q. Nurcombe V. Cool S. M. van Blitterswijk C. A. de Boer J. LaPointe V. L. S. (2017). The components of bone and what they can teach us about regeneration. Materials. 11:E14. 10.3390/ma11010014 29271933 PMC5793512

[B30] Lin H. Cheng A. W. Alexander P. G. Beck A. M. Tuan R. S. (2014a). Cartilage tissue engineering application of injectable gelatin hydrogel with *in situ* visible-light-activated gelation capability in both air and aqueous solution. Tissue Eng. A 20, 2402–2411. 10.1089/ten.tea.2013.0642 24575844 PMC4161187

[B31] Lin H. Lozito T. P. Alexander P. G. Gottardi R. Tuan R. S. (2014b). Stem cell-based microphysiological osteochondral system to model tissue response to interleukin-1β. Mol. Pharm. 11, 2203–2212. 10.1021/mp500136b 24830762 PMC4086740

[B32] Lin H. Tang Y. Lozito T. P. Oyster N. Wang B. Tuan R. S. (2019). Efficient *in vivo* bone formation by BMP-2 engineered human mesenchymal stem cells encapsulated in a projection stereolithographically fabricated hydrogel scaffold. Stem Cell Res. Ther. 10:254. 10.1186/s13287-019-1350-6 31412905 PMC6694509

[B33] Lou Y. R. Toh T. C. Tee Y. H. Yu H. (2017). 25-Hydroxyvitamin D3 induces osteogenic differentiation of human mesenchymal stem cells. Sci. Rep. 7:42816. 10.1038/srep42816 28211493 PMC5314335

[B34] Luu H. H. Song W. X. Luo X. Manning D. Luo J. Deng Z. L.. (2007). Distinct roles of bone morphogenetic proteins in osteogenic differentiation of mesenchymal stem cells. J. Orthop. Res. 25, 665–677. 10.1002/jor.20359 17290432

[B35] Malfait A. M. Liu R. Q. Ijiri K. Komiya S. Tortorella M. D. (2002). Inhibition of ADAM-TS4 and ADAM-TS5 prevents aggrecan degradation in osteoarthritic cartilage. J. Biol. Chem. 277, 22201–22208. 10.1074/jbc.M200431200 11956193

[B36] Mastbergen S. C. Bijlsma J. W. Lafeber F. P. (2005). Selective COX-2 inhibition is favorable to human early and late-stage osteoarthritic cartilage: a human *in vitro* study. Osteoarthr. Cartil. 13, 519–526. 10.1016/j.joca.2005.02.004 15922186

[B37] Mastbergen S. C. Jansen N. W. Bijlsma J. W. Lafeber F. P. (2006). Differential direct effects of cyclo-oxygenase-1/2 inhibition on proteoglycan turnover of human osteoarthritic cartilage: an *in vitro* study. Arthritis Res. Ther. 8:R2. 10.1186/ar1846 16356188 PMC1526565

[B38] Miyazono K. Maeda S. Imamura T. (2005). BMP receptor signaling: transcriptional targets, regulation of signals, and signaling cross-talk. Cytokine Growth Factor Rev. 16, 251–263. 10.1016/j.cytogfr.2005.01.009 15871923

[B39] Morales T. I. Joyce M. E. Sobel M. E. Danielpour D. Roberts A. B. (1991). Transforming growth factor-beta in calf articular cartilage organ cultures: synthesis and distribution. Arch. Biochem. Biophys. 288, 397–405. 10.1016/0003-9861(91)90212-2 1716871

[B40] Mosig A. S. (2017). Organ-on-chip models: new opportunities for biomedical research. Future Sci OA 3:FSO130. 10.4155/fsoa-2016-0038 28670461 PMC5481808

[B41] Mueller M. B. Fischer M. Zellner J. Berner A. Dienstknecht T. Prantl L.. (2010). Hypertrophy in mesenchymal stem cell chondrogenesis: effect of TGF-beta isoforms and chondrogenic conditioning. Cells Tissues Organs 192, 158–166. 10.1159/000313399 20407224 PMC2968769

[B42] Mueller M. B. Tuan R. S. (2008). Functional characterization of hypertrophy in chondrogenesis of human mesenchymal stem cells. Arthritis Rheum. 58, 1377–1388. 10.1002/art.23370 18438858 PMC3612425

[B43] Murphy G. Lee M. H. (2005). What are the roles of metalloproteinases in cartilage and bone damage? Ann Rheum Dis. 64 (Suppl. 4), iv44–iv47. 10.1136/ard.2005.042465 16239386 PMC1766921

[B44] Nakamura H. Masuko K. Yudoh K. Kato T. Nishioka K. (2007). Effects of celecoxib on human chondrocytes–enhanced production of chemokines. Clin. Exp. Rheumatol. 25, 11–16. 17417984

[B45] Nakata K. Hanai T. Take Y. Osada T. Tsuchiya T. Shima D. (2018). Disease-modifying effects of COX-2 selective inhibitors and non-selective NSAIDs in osteoarthritis: a systematic review. Osteoarthr. Cartil. 26, 1263–1273. 10.1016/j.joca.2018.05.021 29890262

[B46] Nejadnik H. Diecke S. Lenkov O. D. Chapelin F. Donig J. Tong X.. (2015). Improved approach for chondrogenic differentiation of human induced pluripotent stem cells. Stem Cell Rev. 11, 242–253. 10.1007/s12015-014-9581-5 25578634 PMC4412587

[B47] Pan J. Zhou X. Li W. Novotny J. E. Doty S. B. Wang L. (2009). *In situ* measurement of transport between subchondral bone and articular cartilage. J. Orthop. Res. 27, 1347–1352. 10.1002/jor.20883 19360842 PMC2748158

[B48] Priam S. Bougault C. Houard X. Gosset M. Salvat C. Berenbaum F.. (2013). Identification of soluble 14-3-3 as a novel subchondral bone mediator involved in cartilage degradation in osteoarthritis. Arthritis Rheum. 65, 1831–1842. 10.1002/art.37951 23552998

[B49] Qu X. Q. Wang W. J. Tang S. S. Liu Y. Wang J. L. (2015). Correlation between interleukin-6 expression in articular cartilage bone and osteoarthritis. Genet. Mol. Res. 14, 14189–14195. 10.4238/2015.November.13.2 26600476

[B50] Raynauld J. P. Martel-Pelletier J. Beaulieu A. Bessette L. Morin F. Choquette D.. (2010). An open-label pilot study evaluating by magnetic resonance imaging the potential for a disease-modifying effect of celecoxib compared to a modelized historical control cohort in the treatment of knee osteoarthritis. Semin. Arthritis Rheum. 40, 185–192. 10.1016/j.semarthrit.2009.10.003 20132966

[B51] Rimando M. G. Wu H. H. Liu Y. A. Lee C. W. Kuo S. W. Lo Y. P.. (2016). Glucocorticoid receptor and histone deacetylase 6 mediate the differential effect of dexamethasone during osteogenesis of mesenchymal stromal cells (MSCs). Sci. Rep. 6:37371. 10.1038/srep37371 27901049 PMC5128810

[B52] Rosenthal N. Brown S. (2007). The mouse ascending: perspectives for human-disease models. Nat. Cell Biol. 9, 993–999. 10.1038/ncb437 17762889

[B53] Sanchez C. Deberg M. A. Piccardi N. Msika P. Reginster J. Y. Henrotin Y. E. (2005). Osteoblasts from the sclerotic subchondral bone downregulate aggrecan but upregulate metalloproteinases expression by chondrocytes. this effect is mimicked by interleukin-6,−1β and oncostatin M pre-treated non-sclerotic osteoblasts. Osteoarthr. Cartil. 13, 979–987. 10.1016/j.joca.2005.03.008 16243232

[B54] Sanchez C. Horcajada M. N. Membrez Scalfo F. Ameye L. Offord E. Henrotin Y. (2015). Carnosol inhibits pro-inflammatory and catabolic mediators of cartilage breakdown in human osteoarthritic chondrocytes and mediates cross-talk between subchondral bone osteoblasts and chondrocytes. PLoS ONE 10:e0136118. 10.1371/journal.pone.0136118 26292290 PMC4546401

[B55] Sanchez C. Mateus M. M. Defresne M. P. Crielaard J. M. Reginster J. Y. Henrotin Y. E. (2002). Metabolism of human articular chondrocytes cultured in alginate beads. longterm effects of interleukin 1beta and nonsteroidal antiinflammatory drugs. J Rheumatol 29, 772–782. 11950021

[B56] Sarzi-Puttini P. Cimmino M. A. Scarpa R. Caporali R. Parazzini F. Zaninelli A.. (2005). Osteoarthritis: an overview of the disease and its treatment strategies. Semin. Arthritis Rheum. 35(1 Suppl. 1), 1–10. 10.1016/j.semarthrit.2005.01.013 16084227

[B57] Sekiya I. Colter D. C. Prockop D. J. (2001). BMP-6 enhances chondrogenesis in a subpopulation of human marrow stromal cells. Biochem. Biophys. Res. Commun. 284, 411–418. 10.1006/bbrc.2001.4898 11394894

[B58] Seok J. Warren H. S. Cuenca A. G. Mindrinos M. N. Baker H. V. Xu W.. (2013). Genomic responses in mouse models poorly mimic human inflammatory diseases. Proc. Natl. Acad. Sci. U.S.A. 110, 3507–3512. 10.1073/pnas.1222878110 23401516 PMC3587220

[B59] Shanks N. Greek R. Greek J. (2009). Are animal models predictive for humans? Philos. Ethics Humanit. Med. 4:2. 10.1186/1747-5341-4-2 19146696 PMC2642860

[B60] Sharma A. R. Jagga S. Lee S. S. Nam J. S. (2013). Interplay between cartilage and subchondral bone contributing to pathogenesis of osteoarthritis. Int. J. Mol. Sci. 14, 19805–19830. 10.3390/ijms141019805 24084727 PMC3821588

[B61] Shintani N. Siebenrock K. A. Hunziker E. B. (2013). TGF-ss1 enhances the BMP-2-induced chondrogenesis of bovine synovial explants and arrests downstream differentiation at an early stage of hypertrophy. PLoS ONE 8:e53086. 10.1371/journal.pone.0053086 23301025 PMC3536810

[B62] Sophia Fox A. J. Bedi A. Rodeo S. A. (2009). The basic science of articular cartilage: structure, composition, and function. Sports Health 1, 461–468. 10.1177/1941738109350438 23015907 PMC3445147

[B63] Stender M. E. Regueiro R. A. Ferguson V. L. (2017). A poroelastic finite element model of the bone-cartilage unit to determine the effects of changes in permeability with osteoarthritis. Comput. Methods Biomech. Biomed. Eng. 20, 319–331. 10.1080/10255842.2016.1233326 27635796

[B64] Su S. C. Tanimoto K. Tanne Y. Kunimatsu R. Hirose N. Mitsuyoshi T.. (2014). Celecoxib exerts protective effects on extracellular matrix metabolism of mandibular condylar chondrocytes under excessive mechanical stress. Osteoarthr. Cartil. 22, 845–851. 10.1016/j.joca.2014.03.011 24721459

[B65] Subramaniam M. Colvard D. Keeting P. E. Rasmussen K. Riggs B. L. Spelsberg T. C. (1992). Glucocorticoid regulation of alkaline phosphatase, osteocalcin, and proto-oncogenes in normal human osteoblast-like cells. J. Cell. Biochem. 50, 411–424. 10.1002/jcb.240500410 1469072

[B66] Tetlow L. C. Adlam D. J. Woolley D. E. (2001). Matrix metalloproteinase and proinflammatory cytokine production by chondrocytes of human osteoarthritic cartilage: associations with degenerative changes. Arthritis Rheum. 44, 585–594. 10.1002/1529-0131(200103)44:3&lt;585::AID-ANR107&gt;3.0.CO;2-C 11263773

[B67] Tsutsumi R. Ito H. Hiramitsu T. Nishitani K. Akiyoshi M. Kitaori T.. (2008). Celecoxib inhibits production of MMP and NO via down-regulation of NF-kappaB and JNK in a PGE2 independent manner in human articular chondrocytes. Rheumatol. Int. 28, 727–736. 10.1007/s00296-007-0511-6 18080123

[B68] Tuli R. Tuli S. Nandi S. Wang M. L. Alexander P. G. Haleem-Smith H.. (2003). Characterization of multipotential mesenchymal progenitor cells derived from human trabecular bone. Stem Cells 21, 681–693. 10.1634/stemcells.21-6-681 14595128

[B69] van der Kraan P. M. (2017). The changing role of TGFβ in healthy, ageing and osteoarthritic joints. Nat. Rev. Rheumatol. 13, 155–163. 10.1038/nrrheum.2016.219 28148919

[B70] Wang W. Rigueur D. Lyons K. M. (2014). TGFβ signaling in cartilage development and maintenance. Birth Defects Res. C Embryo Today 102, 37–51. 10.1002/bdrc.21058 24677722 PMC4267887

[B71] Xu X. Zheng L. Bian Q. Xie L. Liu W. Zhen G.. (2015). Aberrant activation of TGF-β in subchondral bone at the onset of rheumatoid arthritis joint destruction. J. Bone Miner. Res. 30, 2033–2043. 10.1002/jbmr.2550 25967237 PMC4809636

[B72] Yang X. Chen L. Xu X. Li C. Huang C. Deng C. X. (2001). TGF-beta/Smad3 signals repress chondrocyte hypertrophic differentiation and are required for maintaining articular cartilage. J. Cell Biol. 153, 35–46. 10.1083/jcb.153.1.35 11285272 PMC2185521

[B73] Yoon B. S. Ovchinnikov D. A. Yoshii I. Mishina Y. Behringer R. R. Lyons K. M. (2005). Bmpr1a and Bmpr1b have overlapping functions and are essential for chondrogenesis *in vivo*. Proc. Natl. Acad. Sci. U.S.A. 102, 5062–5067. 10.1073/pnas.0500031102 15781876 PMC555995

[B74] Yoon B. S. Pogue R. Ovchinnikov D. A. Yoshii I. Mishina Y. Behringer R. R.. (2006). BMPs regulate multiple aspects of growth-plate chondrogenesis through opposing actions on FGF pathways. Development 133, 4667–4678. 10.1242/dev.02680 17065231

[B75] Yuan X. L. Meng H. Y. Wang Y. C. Peng J. Guo Q. Y. Wang A. Y.. (2014). Bone-cartilage interface crosstalk in osteoarthritis: potential pathways and future therapeutic strategies. Osteoarthr. Cartil. 22, 1077–1089. 10.1016/j.joca.2014.05.023 24928319

[B76] Zhang X. Ziran N. Goater J. J. Schwarz E. M. Puzas J. E. Rosier R. N.. (2004). Primary murine limb bud mesenchymal cells in long-term culture complete chondrocyte differentiation: TGF-beta delays hypertrophy and PGE2 inhibits terminal differentiation. Bone 34, 809–817. 10.1016/j.bone.2003.12.026 15121012

[B77] Zhang Y. Jordan J. M. (2010). Epidemiology of osteoarthritis. Clin. Geriatr. Med. 26, 355–369. 10.1016/j.cger.2010.03.001 20699159 PMC2920533

[B78] Zhen G. Wen C. Jia X. Li Y. Crane J. L. Mears S. C.. (2013). Inhibition of TGF-β signaling in mesenchymal stem cells of subchondral bone attenuates osteoarthritis. Nat. Med. 19, 704–712. 10.1038/nm.3143 23685840 PMC3676689

[B79] Zweers M. C. de Boer T. N. van Roon J. Bijlsma J. W. Lafeber F. P. Mastbergen S. C. (2011). Celecoxib: considerations regarding its potential disease-modifying properties in osteoarthritis. Arthritis Res. Ther. 13:239. 10.1186/ar3437 21955617 PMC3308065

