# Supplementary material for: Osteochondral Tissue Chip Derived From iPSCs: Modeling OA Pathologies and Testing Drugs
Source: Front Bioeng Biotechnol. 2019 Dec 17;7:411. doi: 10.3389/fbioe.2019.00411 (PMC6930794; doi:10.3389/fbioe.2019.00411)
Supplement: Supplementary file 1 [file Data_Sheet_1.docx]

**Supplemental Figures**

**
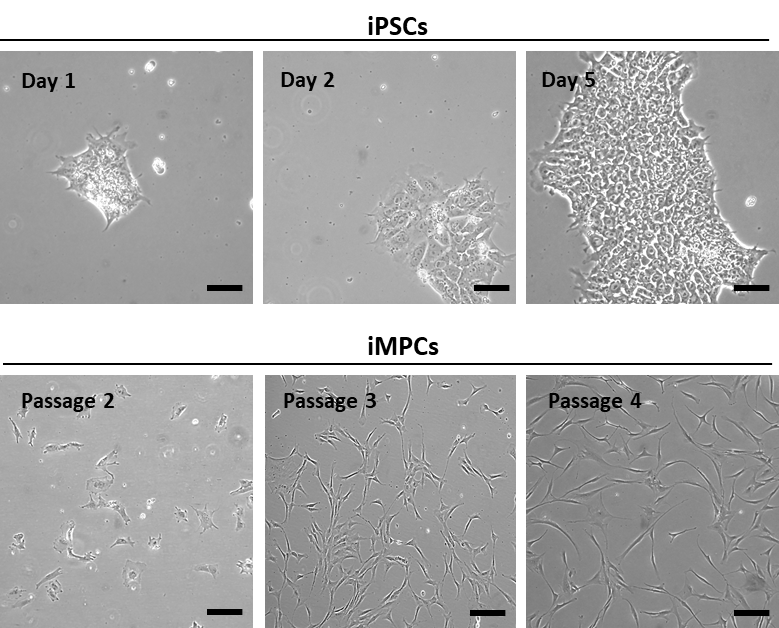
**

**Figure S1.** Morphology of the iPSCs and iMPCs at different passages. Bar = 100 µm.


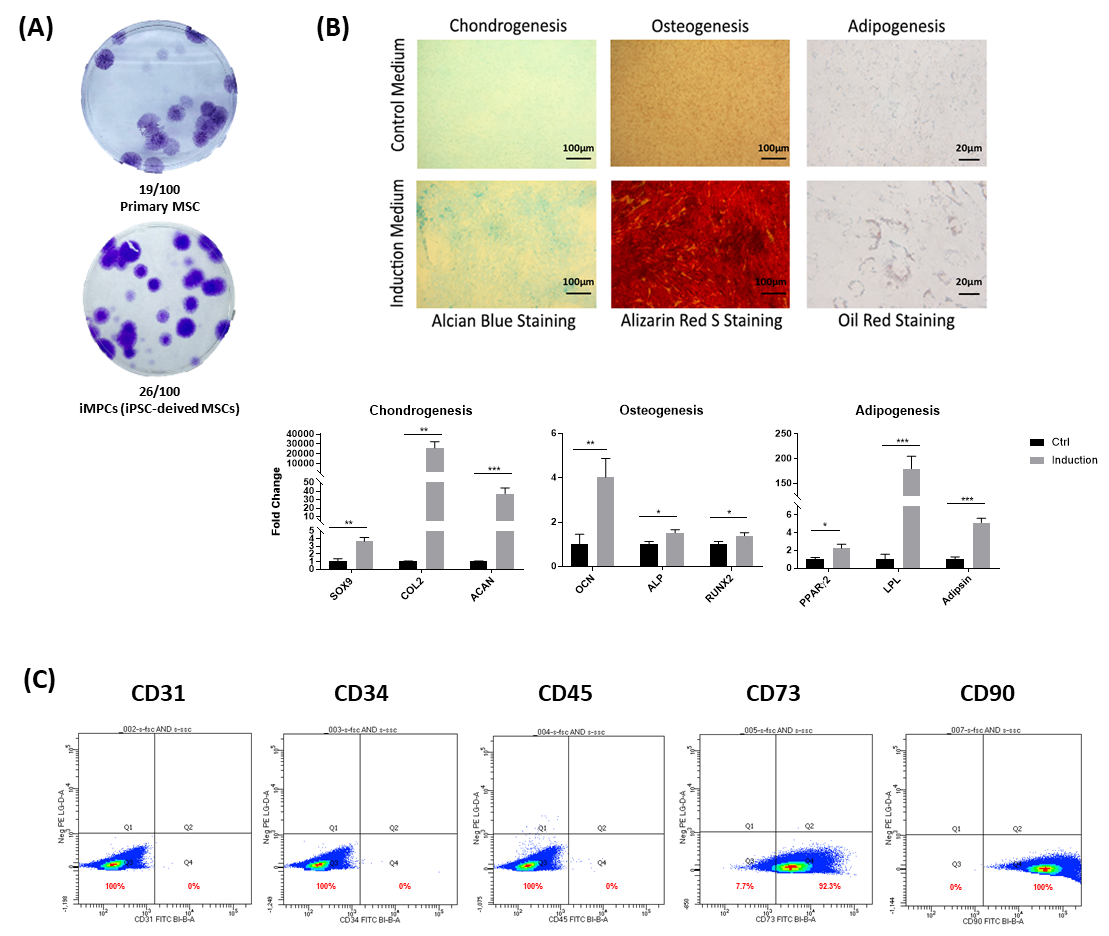


**Figure S2.** MSC-like characteristics of iMPCs. (A) CFU assay. iMPCs have similar colony forming ability compared to primary MSCs. (B) Tri-lineage differentiation. Compared to unstimulated control, iMPCs showed significant tissue-specific gene expression and displayed positive staining for Alcian Blue, Alizarin Red S, and Oil Red Staining respectively, confirming their ability to undergo chondrogenic, osteogenic and adipogenic differentiation. Gene expression levels of Ctrl are normalized to 1. Values are presented as mean ± SD (N = 4 per group: *, p < 0.05; **, p < 0.01; ***, p< 0.001). (C) Flow cytometric analysis. iMPCs displayed similar cell surface marker profile as primary human MSCs, i.e., negative for CD31, CD34 and CD45, and positive for CD73 and CD90.


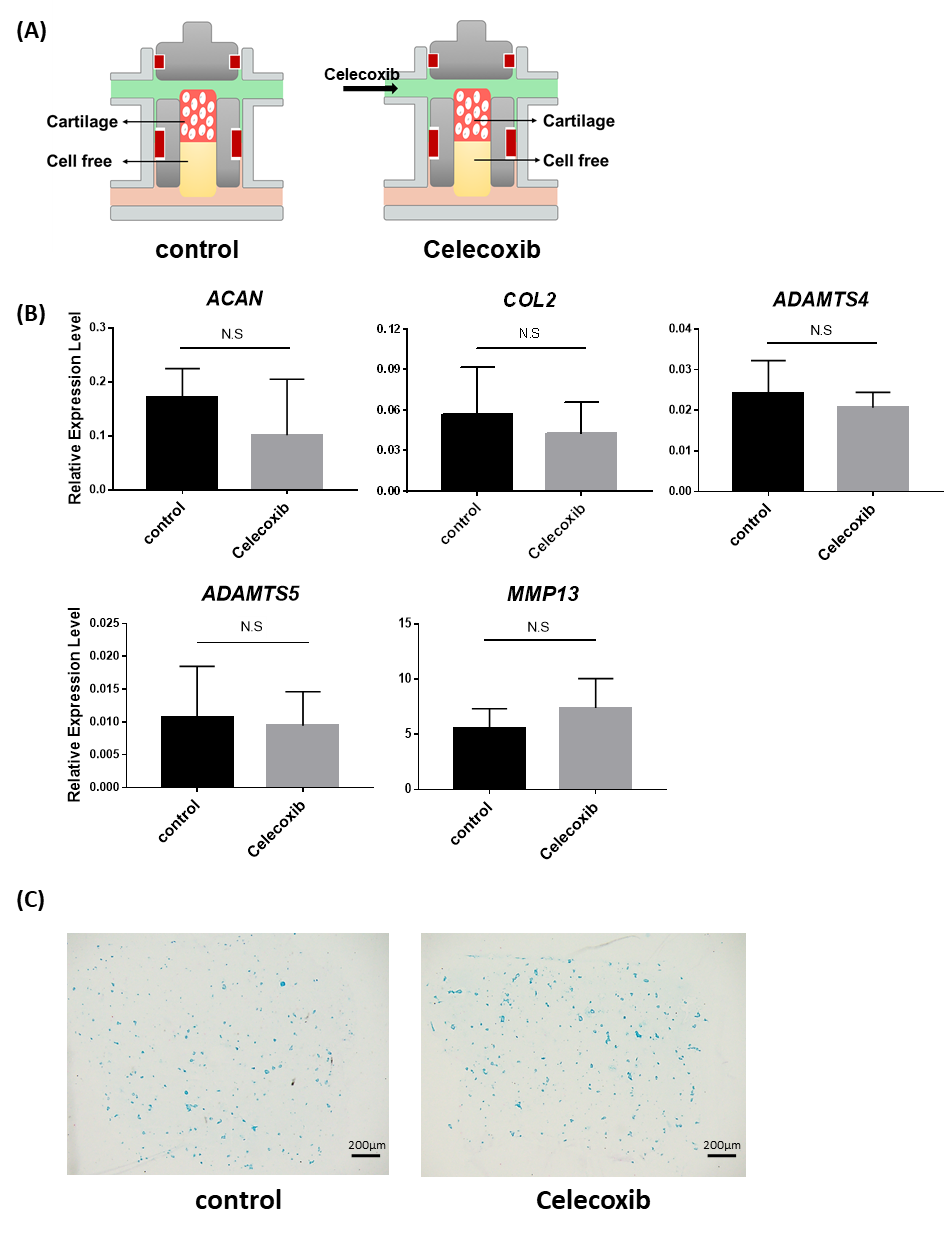


**Figure S3.** Lack of toxicity of Celecoxib treatment of engineered cartilage. (A) Schematic of control and celecoxib treatment groups. (B) Expression of chondrocyte-associated genes. After Celecoxib treatment, no significant changes in the expression levels of all tested genes were observed. (C) Alcian Blue staining. No significant change in cartilage matrix was seen after Celecoxib treatment.

**Table S1. Sequences of primers used for quantitative PCR**

| **Gene** | **Forward Primer (5'-3')** | **Reverse Primer (5'-3')** |
| --- | --- | --- |
| *RPL13A* | CATAGGAAGCTGGGAGCAAG | GCCCTCCAATCAGTCTTCTG |
| *GAPDH* | GGAGCGAGATCCCTCCAAAAT | GGCTGTTGTCATACTTCTCATGG |
| *SOX9* | GGCGGAGGAAGTCGGTGAAGAA | GCTCATGCCGGAGGAGGAGTGT |
| *COL2* | GGATGGCTGCACGAAACATACCGG | CAAGAAGCAGACCGGCCCTATG |
| *ACAN* | AGTCACACCTGAGCAGCATC | AGTTCTCAAATTGCATGGGGTGTC |
| *COL10* | CCCTCTTGTTAGTGCCAACC | AGATTCCAGTCCTTGGGTCA |
| *MMP13* | ATGCAGTCTTTCTTCGGCTTAG | ATGCCATCGTGAAGTCTGGT |
| *RUNX2* | GTGATAAATTCAGAAGGGAGG | CTTTTGCTAATGCTTCGTGT |
| *OCN* | TCACACTCCTCGCCCTATTG | GAAGAGGAAAGAAGGGTGCC |
| *ALP* | ATCTTTGGTCTGGCCCCCATG | AGTCCACCATGGAGACATTCTCTC |
| *BSP2* | CGAATACACGGGCGTCAATG | GTAGCTGTACTCATCTTCATAGGC |
| *TNF-α* | CCTCTCTCTAATCAGCCCTCTG | GAGGACCTGGGAGTAGATGAG |
| *IL-1β* | ATGATGGCTTATTACAGTGGCAA | GTCGGAGATTCGTAGCTGGA |
| *IL-6* | ACTCACCTCTTCAGAACGAATTG | CCATCTTTGGAAGGTTCAGGTTG |
| *MMP1* | AAAATTACACGCCAGATTTGCC | GGTGTGACATTACTCCAGAGTTG |
| *MMP2* | TACAGGATCATTGGCTACACACC | GGTCACATCGCTCCAGACT |
| *MMP3* | CTGGACTCCGACACTCTGGA | CAGGAAAGGTTCTGAAGTGACC |
| *MMP9* | TGTACCGCTATGGTTACACTCG | GGCAGGGACAGTTGCTTCT |
| *ADAMTS-4* | CTGGCACCTACCTGACTGG | GTAACACGCCTAACAGGGCT |
| *ADAMTS-5* | ATCACCCAATGCCAAGG | AGCAGAGTAGGAGACAAC |
| *TGF-β3* | GGAAAACACCGAGTCGGAATAC | GCGGAAAACCTTGGAGGTAAT |
