# Supplementary material for: Osteochondral Tissue Chip Derived From iPSCs: Modeling OA Pathologies and Testing Drugs
Source: Front Bioeng Biotechnol. 2019 Dec 17;7:411. doi: 10.3389/fbioe.2019.00411 (PMC6930794; doi:10.3389/fbioe.2019.00411)
Supplement: Supplementary file 2 [file Data_Sheet_2.docx]

|  | Title | GEO accession |
| --- | --- | --- |
| Paper | Osteochondral Tissue Chip Derived from iPSCs: Modeling OA  pathologies and Testing Drugs | GSE140337 |
| Figure 1 | Real-time quantitative PCR analysis of chondrogenic markers  in iMPC with different treatments | GSE140330 |
| Figure 2 | Real-time quantitative PCR analysis of osteogenic markers  in iMPC with different treatments | GSE140331 |
| Figure 3 | Real-time quantitative PCR analysis of phenotype of  osteochondral tissue chip | GSE140332 |
| Figure 4 | Real-time quantitative PCR analysis of chondrosupportive  activity of bone component in the engineered osteochondral tissue | GSE140334 |
| Figure 5 | Real-time quantitative PCR analysis of OC-C and CH under  osteoarthritic condition | GSE140335 |
| Figure 6 | Real-time quantitative PCR analysis of celecoxib treatment  on OC tissue chip | GSE140336 |
